# Antithyroid drug induced syndrome that lies in between ANCA vasculitis and lupus-like syndrome in a 40-year-old female with Graves’ disease under methimazole therapy: A Case report

**DOI:** 10.31138/mjr.29.1.52

**Published:** 2018-03-19

**Authors:** Eleni Nikitopoulou, Sousana Gazi

**Affiliations:** Rheumatology Clinic, General Hospital KAT, Kifissia, Athens, Greece

**Keywords:** polyarthritis, methimazole, antithyroid drug, ANCA, vasculitis, lupus-like syndrome

## Abstract

We report the case of a 40-year-old Greek female with symptoms of polyarthritis, pruritic rash and positive p-ANCA antibodies, undergoing treatment with Methimazole therapy for Graves’ disease. The rash and the arthritis symptoms promptly disappeared after withdrawal of methimazole, but p-ANCA antibodies remained positive for 6 weeks. By the time that p-ANCA became negative, anti-dsDNA antibodies presented and remained at high titers for 3 months, with no clinical or specific organ disease symptoms. The patient was under close monitoring for the case of potentially life-threating vasculitis of the lung or the kidney and was treated with methylprednisolone. We diagnosed the patient with Antithyroid drug Syndrome, which in our patient presented with arthritis symptoms and had serological features which are commonly found to Antithyroid drug pANCA vasculitis and Antithyroid drug lupus-like syndrome. Physician’s awareness is essential for the diagnosis and treatment of this syndrome in clinical practice, taking into consideration the high frequency of the use of antithyroid agents.

A 40-year-old female was referred to our clinic with symptoms of asymmetric polyarthritis of the upper and lower extremities, morning stiffness lasting 1 hour and constitutional symptoms such as generalised arthralgias and myalgias, fatigue and malaise for a period of 15 days prior the first examination. She presented with a pruritic rash of the eyebrows, the neck and the trunk. She had no history of allergies, or collagen diseases and she abstained from smoking and was a social drinker. She was mother of two children and reported no miscarriages. Her family history did not include rheumatic diseases. Four years ago, she had been diagnosed with Graves’ disease and had been treated ever since with antithyroid drugs. Specifically, the first 2 years she received carbimazole, then, for 6 months she was treated with propylthiouracil and subsequently, over the past 11 months she was receiving methimazole 20mg/day.

Physical examination showed no fever, normal vital signs, and normal findings from the lungs, cardiovascular, gastrointestinal and nervous system. She had a pruritic exfoliative rash over the eyebrow area, the neck and upper trunk and aphthous lesions in oral mucosa. There was obvious painful oedema of the left hand and the left foot with redness of the overlying skin. In addition, restriction of the active and passive movements and synovitis of the left wrist, the second, a third and fourth metacarpophalangeal joint of the left hand and of the left ankle were detected.

At that point, the x-rays of the lungs, wrists and metacarpophalangeal joints were normal. The laboratory testing revealed low total white blood cell count at 2920cells/ul with normal differential, hematocrit at 33,7%, elevated erythrocyte sedimentation rate (54mm/hr), CRP within normal levels, normal levels of thyroid stimulation hormone and positive results for antithyroid peroxidase antibody and antithyroglobulin antibody. The serological testing showed positive ANA at 1:640 (normal,<1:80), positive ANCA (1:160; normal,<1:20) including positive myeloperoxidase (MPO) p-ANCA and negative protein-ase (PR3) ANCA, low complement C3 (31mg/dl;normal range, 90–180), and C4 (2,6mg/dl; normal range 10–40). On the basis of these findings, the patient was diagnosed with antithyroid drug induced vasculitis syndrome as an adverse reaction to the methimazole treatment, manifesting as skin rash, inflammatory arthritis, constitutional symptoms accompanied by a specific serological profile. Therefore, we immediately discontinued the administration of methimazole and set the patient under close monitoring for the case of potentially life-threating vasculitis of lung or kidney and prescribed prednisolone at starting dose 16mg per day. The patient’s symptoms resolved quickly. The rash completely disappeared within 36 hours, and did not allow us to make a skin biopsy. In addition, the pain and swelling in the affected joints settled within 3 days. The resolution of symptoms shortly after the discontinuation of methimazole, reinforced our hypothesis that this was a case of antithyroid drug induced syndrome. Follow-up evaluation at 6 weeks revealed p-ANCA negative, WBC count 5000/ul, and low C3 (35mg/dl) and C4 (1mg/dl).

Two weeks later, anti-dsDNA antibodies were positive at 1580 units (normal <20) while C4 complement was still at the lower normal levels. Additional tests were performed towards systemic lupus erythematosus disease spectrum, including anti-Sm antibodies, anti-Ro antibodies, anti-La antibodies, direct Coombs test, anti-cardiolipin antibodies, anti-b2GPI antibodies and lupus anticoagulant, all of which were negative. At 6 months follow up, the patient was in good health, and anti-ds-DNA antibodies were negative.

## DISCUSSION

Drug-induced vasculitis is an adverse reaction of antithyroid treatment. It can manifest with symptoms that range from less specific, such as skin rash, arthritis and constitutional symptoms, to systemic involvement and is associated with ANCA-positivity.^1,2^ Since 1992, over a hundred cases of patients treated for Graves’ disease have been reported with antithyroid drug vasculitis.^3,8^ Some researchers suggest that life-threatening vasculitis might be developed in patients with systemic non-specific syndrome when the causal antithyroid drug is not withdrawn timely.^2,3,9^ The majority of these patients having MPO-ANCA while arthralgias and arthritis are commonly reported as initial symptoms.^1–9^

On the other hand, drug-induced lupus has been described in patients treated with antithyroid agents and manifests also with less specific symptoms such as rashes and arthralgias. Some researchers rely on the presence of specific autoantibodies to make a distinction of lupus-like syndrome from vasculitis like syndrome.^7,10,11^ However, it is quite often that drug-induced vasculitis cannot be clearly distinguished from lupus-like syndrome.^4–6,8^ It has been described that repeated treatment with antithyroid agents and other causative agents can lead to the development of various autoantibodies including MPO-ANCA, antinuclear antibodies, anti-dsDNA antibodies and anti-cardiolipin antibodies in up to 10% of patients.^12^

The pathogenetic mechanism is not fully understood and might be multifactorial: antithyroid agents given in genetic predisposed patients along with environmental trigger factors such as infections, can induce neutrophil activation and apoptosis. Neutrophil apoptosis, in the absence of priming, is associated with translocation of ANCA antigens to the cell surface inducing the production of ANCA antibodies. In the same time, the apoptotic blebs of neutrophils and the NETs formation can be a source of immunogens for the production of various autoantibodies such as anti-dsDNA, antiSSA, and anti-b2GPI.^9^

Many researchers suggest that it is irrelevant to distinguish between antithyroid drug-induced lupus-like syndrome and antithyroid drug-induced ANCA vasculitis syndrome, irrespective of the serologic profile is.^8,13^ Although, others make a clear distinction between these conditions and claim that lupus like syndrome has a better outcome.^3,8,10^ In the majority of reported cases with lupus-like or vasculitis-like syndrome caused by antithyroid agents, the most important factors that define the final diagnosis are the clinical features and the final organ impairment, while laboratory results play a secondary and supportive role. ^8,10,13^ Antithyroid agents can cause an autoimmune syndrome which lies in between vasculitis like and lupus like syndrome, while there are no specific diagnostic criteria at the initiation of each case or other markers, that we can rely on to prognose the final outcome, no matter how we name and categorize it at the very end.^8,11^

The present case appears to be an antithyroid drug induced syndrome, in the general context of antithyroid drug induced vasculitis and lupus-like syndrome. Our diagnosis was based on the dramatic improvement of musculoskeletal and skin symptoms after the withdrawal of methimazole, the lack of serious vasculitis complications (e.g., nephritis, lung involvement) and the short period that ANCA and anti-dsDNA antibodies were present.^9^ Given the unpredictable and potentially life-threating vasculitis could be induced by such therapy, it is crucial that clinicians should be aware of this condition, recognize it and promptly withdraw the causative antithyroid agent.
